# Cross-protection against European swine influenza viruses in the context of infection immunity against the 2009 pandemic H1N1 virus: studies in the pig model of influenza

**DOI:** 10.1186/s13567-015-0236-6

**Published:** 2015-09-24

**Authors:** Yu Qiu, Karl De hert, Kristien Van Reeth

**Affiliations:** Laboratory of Virology, Faculty of Veterinary Medicine, Ghent University, Salisburylaan 133, B-9820 Merelbeke, Belgium

## Abstract

Pigs are natural hosts for the same influenza virus subtypes as humans and are a valuable model for cross-protection studies with influenza. In this study, we have used the pig model to examine the extent of virological protection between a) the 2009 pandemic H1N1 (pH1N1) virus and three different European H1 swine influenza virus (SIV) lineages, and b) these H1 viruses and a European H3N2 SIV. Pigs were inoculated intranasally with representative strains of each virus lineage with 6- and 17-week intervals between H1 inoculations and between H1 and H3 inoculations, respectively. Virus titers in nasal swabs and/or tissues of the respiratory tract were determined after each inoculation. There was substantial though differing cross-protection between pH1N1 and other H1 viruses, which was directly correlated with the relatedness in the viral hemagglutinin (HA) and neuraminidase (NA) proteins. Cross-protection against H3N2 was almost complete in pigs with immunity against H1N2, but was weak in H1N1/pH1N1-immune pigs. In conclusion, infection with a live, wild type influenza virus may offer substantial cross-lineage protection against viruses of the same HA and/or NA subtype. True heterosubtypic protection, in contrast, appears to be minimal in natural influenza virus hosts. We discuss our findings in the light of the zoonotic and pandemic risks of SIVs.

**Electronic supplementary material:**

The online version of this article (doi:10.1186/s13567-015-0236-6) contains supplementary material, which is available to authorized users.

## Introduction

Swine influenza viruses (SIVs) are important for the swine industry and as zoonotic agents. Moreover, they can lead to the emergence of novel pandemic influenza viruses for humans. In Europe, four lineages of SIV are enzootic in swine populations. An H1N1 virus of wholly avian origin became established in European swine in 1979 [1]. In the mid 1980s, this H1N1 virus reassorted with descendants of the 1968 Hong Kong human pandemic H3N2 virus [2,3]. The resulting H3N2 SIV lineage has human-like hemagglutinin (HA) and neuraminidase (NA) genes and avian-like internal genes. The third lineage, H1N2, was first reported in 1994, and is a reassortant virus that retains most of the genome of the H3N2 SIV, but has acquired an H1 gene from human seasonal viruses from the 1980s [4,5]. The 2009 pandemic H1N1 (pH1N1) virus is a reassortant with the NA and matrix (M) genes derived from the European avian-like H1N1 SIV and the remaining genes from North American triple-reassortant H1 SIVs [6]. The pH1N1 virus was first detected in humans in April 2009 and only later in swine, but it has become widespread in swine worldwide due to large-scale reverse zoonotic transmissions [7]. Thus, while all four SIV lineages have a distinct HA and/or NA, the pH1N1 also has a different set of internal genes compared to the three previously established SIVs. A growing number of reassortants between these four lineages has been reported in recent years, especially between pH1N1 and previously established SIVs [8].

The increasing number of H1 SIV lineages in Europe and other continents, and the geographic differences in the prevailing lineages have spurred interests in the extent of cross-protection between them. Prior infection of pigs with a European avian-like H1N1 SIV largely protects against subsequent infection with the pH1N1 [9], or with a North American triple-reassortant H1N1 SIV [10], despite the absence of cross-reactive serum hemagglutination-inhibition (HI) antibodies against the challenge virus. It remains unknown to what extent prior infection with pH1N1 offers protection against the previously established European H1 SIVs. This question is also of public health concern as the global spread of pH1N1 may generate cross-reactive immunity against some H1 SIVs in the human population, making them less likely candidates for future pandemics.

Apart from cross-protection between variants of the same HA subtype, cross-protection between viruses of different HA subtypes (heterosubtypic protection) has also been described. Heterosubtypic protection has been repeatedly shown in rodents and ferrets [11-15], but only rarely in natural hosts of influenza. In an experimental pig infection study with European SIVs, only 1 out of 5 H1N1-immune pigs tested positive for the H3N2 challenge virus in oropharyngeal swabs, for 1 day only. However, challenge control pigs in that study also had minimal virus titers in oropharyngeal swabs, and nasal swabs or tissues of the respiratory tract were not examined [16]. Epidemiological data support the existence of heterosubtypic immunity in humans that were exposed simultaneously or consecutively to epidemic human seasonal H1N1 and H3N2 viruses [17,18]. Also, the 1957 pandemic H2N2 virus appeared to have a lower disease incidence in adults previously infected with an H1N1 virus [19]. Yet, the significance and importance of heterosubtypic immunity in natural influenza virus hosts remain unclear. In this study, we sought to study cross-protection between a) pH1N1 and various H1 SIVs, and b) these distinct H1 SIVs and H3N2. We use the pig as a natural host for SIVs and a model for influenza in humans.

## Material and methods

### Viruses and their genetic and antigenic relationships

**Figure 1:**
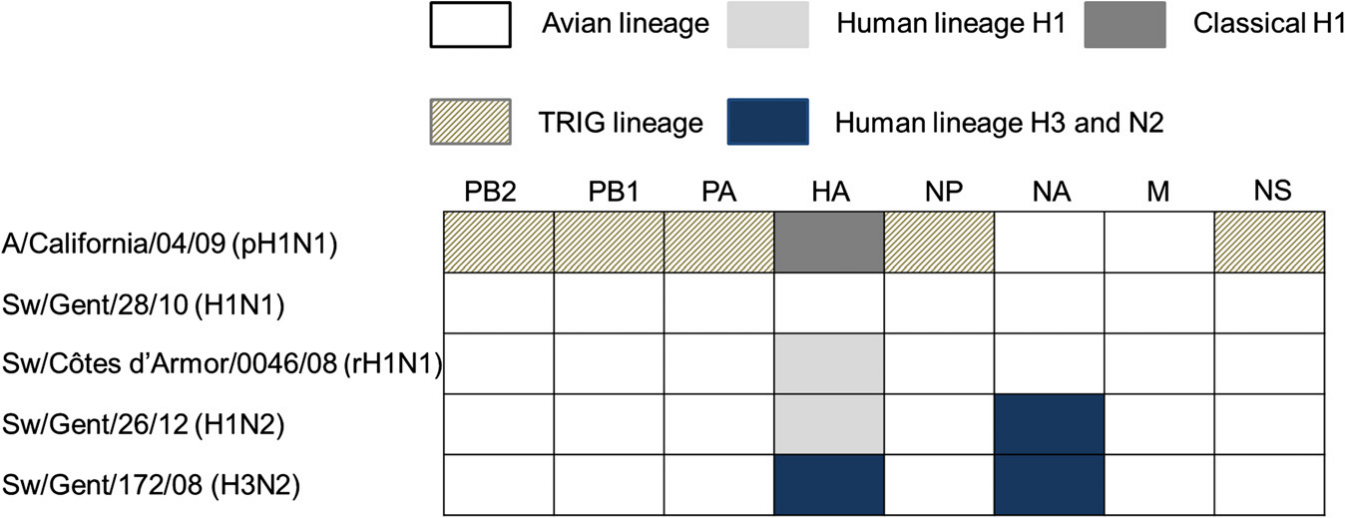
**Genetic constellations of the five viruses used in this study.** Abbreviations: PB2, polymerase basic 2; PB1, polymerase basic 1; PA, polymerase acidic; HA, hemagglutinin; NP, nucleoprotein; NA, neuraminidase; M, matrix; NS, nonstructural; TRIG, triple-reassortant internal genes, which are derived from swine (M, NS and NP), human (PB1) and avian (PB2 and PA) influenza viruses forming a constellation of genes that is well conserved in North American swine influenza viruses.

Viruses for pig inoculation were propagated in embryonated chicken eggs and used at the third or fourth passage. Their genetic constellations are shown in Figure 1. A/California/04/09 is a representative pH1N1, while sw/Gent/28/10 (H1N1), sw/Gent/26/12 (H1N2) and sw/Gent/172/08 (H3N2) are representative for SIVs that are enzootic in Western Europe. Sw/Côtes d’Armor/0046/08 is an occasionally reported reassortant H1N1 (rH1N1) SIV with the H1 derived from the European H1N2 SIV lineage and the N1 from the European H1N1 lineage [20].

The sequences of the HA1 and NA segments of the 5 viruses were available in GenBank (accession numbers FN646093, FN646099, KC142127, KC142128 and KP406524-KP406529). The HA1 and NA segments were compared at the amino acid level using the MEGALIGN program (DNASTAR, Madison, WI, USA). Amino acid differences at putative antigenic sites of the H1 and N2 proteins, as defined previously [21,22], were identified by alignment using MEGA5 software [23]. Antigenic characterization of the 5 viruses was performed by HI, virus-neutralization (VN) and neuraminidase-inhibition (NI) assays, using pig sera collected at 2 weeks after inoculation with each individual virus.

### Experimental design

Forty 6-week-old pigs from an influenza negative farm were randomly assigned to 8 groups (*n* = 5) as shown in Table 1. Each group was housed in a separate biosafety level-2 HEPA-filtered isolation unit. All experiments were authorized by the Ethical and Animal Welfare Committee of the Faculty of Veterinary Medicine, Ghent University. Virus inoculations of pigs were performed intranasally, using 7.0 log_10_ 50% egg infectious doses (EID_50_) of the respective viruses in 3 mL (1.5 mL per nostril). Pigs were unanesthetized and held in a vertical position with the neck stretched. The inoculum was instilled into the middle nasal cavity by insertion of a 15-mm plastic cannula attached to a 5-mL syringe.

**Table 1:** **Experimental design**

	Viruses used for inoculation		
	Experiment 1		Experiment 2
Group	7 weeks^a^	13 weeks^a^	30 weeks^a^
A	pH1N1	pH1N1	H3N2
B	pH1N1	H1N1	H3N2
C	pH1N1	rH1N1	-
D	pH1N1	H1N2	H3N2
E	PBS	H1N1	H3N2
F	PBS	rH1N1	-
G	PBS	H1N2	H3N2
H	PBS	PBS	H3N2

^a^ The age of pigs at the time of virus inoculation.

The first experiment was designed to examine whether infection immunity against pH1N1 offers protection against infection with European H1 SIVs (Table 1). Four groups of pigs (A, B, C and D) were inoculated with pH1N1 virus, and the remaining four groups (E, F, G and H) were mock-inoculated with phosphate-buffered saline (PBS). Six weeks later, the pH1N1-immune pigs were inoculated with the same pH1N1 virus (group A), or with 1 of 3 European H1 SIVs: H1N1 (group B), rH1N1 (group C), or H1N2 (group D). Three groups of influenza naïve pigs served as H1N1 (group E), rH1N1 (group F), or H1N2 (group G) challenge controls. Group H was inoculated again with PBS. To determine virus excretion, nasal swabs for virus titration were collected daily from all pigs from 0–8 days post-primary inoculation and 0–7 days post-secondary inoculation. Blood samples for serology were collected at 0 and 14 days post-primary inoculation, and at 0, 5, 7, 10 and 14 days post-secondary inoculation.

The second experiment aimed at studying the heterosubtypic protection between H1 and H3 viruses (Table 1). Six groups from the first experiment (A, B, D, E, G and H) were inoculated with H3N2, 17 weeks after the secondary inoculation. Nasal swabs for virus titration were collected daily from all pigs from 0–7 days post-tertiary inoculation, or until euthanasia. Two pigs per group were euthanized at 4 days post-tertiary inoculation to examine virus titers of the entire respiratory tract: nasal mucosa respiratory and olfactory regions, tonsil, trachea, apical, cardiac, and diaphragmatic lobes of the left and right lungs, and the accessory lung lobe. Each tissue sample was collected and titrated separately. Blood samples for serology were collected at 0 and 14 days post-tertiary inoculation.

### Virus titration

Sterile nasal swabs (Copan 160C, Copan Italia S.p.A.) were weighed before and after collection to determine virus titers per 100 mg nasal secretions. Swabs from both nostrils were suspended in 1 mL sterile PBS supplemented with antibiotics. Tissues were weighed and homogenized in sterile PBS with antibiotics to obtain 20% (w/v) homogenates. Nasal swab specimens and tissue homogenates were titrated in Madin-Darby canine kidney (MDCK) cells by the 50% tissue culture infectious doses (TCID_50_) assay as described elsewhere [24].

### Serological assays

Serum antibody responses were examined in HI, VN and NI assays, as described elsewhere [25,26]. All sera collected before and at 2 weeks after each inoculation were examined against all 5 viruses in all 3 assays. At 5, 7, 10 and 14 days post-secondary inoculation, additional VN assays against the respective challenge H1 viruses were performed. Antibody titers were expressed as the reciprocal of the highest serum dilution that showed complete inhibition of HA of 4 hemagglutinating units of virus (HI assay), 50% neutralization of 100 TCID_50_ of virus in MDCK cells (VN assay), or 50% reduction of NA activity (NI assay). Starting dilutions were 1:2 in the VN assay, and 1:10 in HI and NI assays.

### Statistics

Nasal virus shedding in each group was quantified by calculation of the area under the curve (AUC). Mann–Whitney tests were used to compare antibody levels between any two experimental groups, and before and after inoculation in each group. Differences were considered significant when *p* < 0.05. GraphPad Prism5 software (GraphPad Software, San Diego, CA, USA) was used for all statistical analyses.

## Results

### Genetic and antigenic relationships between pH1N1 and European SIVs

Genetic relationships between viruses were assessed by comparison of the homology in viral HA1 and NA amino acid sequences (Table 2). Antigenic relationships between viruses were examined in cross-HI, VN and NI assays, using monospecific pig sera (Table 3). The rH1N1 and H1N2 viruses showed 90% amino acid sequence identity in their HA1 segments and cross-reactivity in HI and VN assays, reflecting the same human-like HA lineage of the two viruses. The HA1 segments of H1N1, rH1N1 and H1N2 had a similar homology (71-73%) to the classical H1 of pH1N1. Alignment of HA1 antigenic sites of pH1N1 with those of H1N1, rH1N1 and H1N2 revealed 17, 26 and 27 amino acid differences, respectively (Additional file 1). Cross-reactivity between pH1N1 and other European H1 SIVs was absent in the HI assay, and rare in the VN assay. H3N2 failed to cross-react with any H1 viruses in HI and VN assays.

**Table 2:** **Percent identity of the amino acid sequences of viral hemagglutinin (HA1) and neuraminidase (NA) segments**

Virus	A/California/04/09		Sw/Gent/28/10		Sw/Côtes d’Armor/0046/08		Sw/Gent/26/12		Sw/Gent/172/08	
	HA1	NA	HA1	NA	HA1	NA	HA1	NA	HA1	NA
A/California/04/09 (pH1N1)	100	100								
Sw/Gent/28/10 (H1N1)	73	91	100	100						
Sw/Côtes d’Armor/0046/08 (rH1N1)	72	91	70	97	100	100				
Sw/Gent/26/12 (H1N2)	71	41	69	40	90	40	100	100		
Sw/Gent/172/08 (H3N2)	34	42	35	40	32	39	33	84	100	100

**Table 3:** **Geometric mean antibody titers in hemagglutination-inhibition (HI), virus-neutralization (VN), and neuraminidase-inhibition (NI) assays at 14 days post-inoculation of pigs with various influenza viruses.**

		Antibody titer against														
Virus for inoculation	No. of pigs	A/California/04/09			Sw/Gent/28/10			Sw/Côtes d’Armor/0046/08			Sw/Gent/26/12			Sw/Gent/172/08		
		HI	VN	NI	HI	VN	NI	HI	VN	NI	HI	VN	NI	HI	VN	NI
A/California/04/09 (pH1N1)	20	**89**	**76**	**109**	<10	3	16	<10	9	22	<10	4	<10	<10	<2	<10
Sw/Gent/28/10 (H1N1)	5	<10	3	16	**80**	**384**	**320**	<10	3	40	<10	4	<10	<10	<2	<10
Sw/Côtes d’Armor/0046/08 (rH1N1)	5	<10	2	30	<10	<2	40	**70**	**795**	**211**	20	262	<10	<10	<2	<10
Sw/Gent/26/12 (H1N2)	5	<10	<2	<10	<10	<2	<10	15	114	<10	**127**	**1276**	**46**	<10	<2	15
Sw/Gent/172/08 (H3N2)	3	<10	<2	<10	<10	<2	<10	<10	<2	<10	<10	<2	12	**160**	**347**	**381**

The detection limits were 1:2 in the VN assay, and 1:10 in HI and NI assays. Negative samples were assigned a value of half the minimum detectable titer for the calculation of geometric mean antibody titers. Values in bold are titers against the homologous virus.

The H1N1 and rH1N1 showed 97% amino acid identity in their NAs, which were closely related to the NA of pH1N1 (91% identity), reflecting the same avian-like N1 lineage of the three viruses. Unfortunately, the complete antigenic sites on N1 neuraminidase have not been defined yet. The human-like NAs of H1N2 and H3N2 had 84% amino acid identity and 10 residue differences in antigenic sites (Additional file 2). Consistent with the genetic relatedness, cross-reactivity in the NI assay was observed between viruses of the same avian-like N1 or human-like N2 lineage.

### Experiment 1: protection against challenge with various European H1 SIVs in pigs with infection immunity against pH1N1

After primary inoculation with pH1N1, pigs from groups A, B, C and D had similar mean AUC values (range from 24.9-27.4) and nasal shedding during 6–7 days. The mock-inoculated pigs tested negative for virus at all time points.

**Figure 2:**
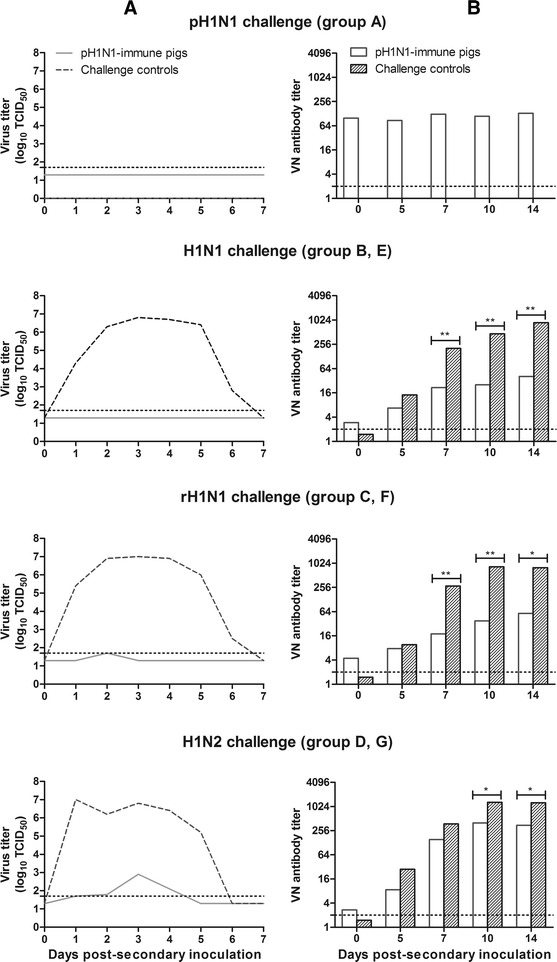
**Virus titers in nasal swabs (A) and virus-neutralizing (VN) antibody titers in serum (B) post-secondary inoculation with various H1 viruses.** Nasal swabs were collected daily from day 0 to 7 post-secondary inoculation to determine virus titers per 100 mg nasal secretions. VN antibody titers were determined against the respective challenge H1 virus on days 0, 5, 7, 10 and 14 post-secondary inoculation. Horizontal dotted lines represent the detection limit of the assay: 1.7 log_10_ TCID_50_ for virus titration, 2 for the VN assay. *p < 0.05 and **p < 0.01, by the Mann–Whitney test.

Figure 2A shows mean virus titers in nasal swabs post-secondary inoculation with pH1N1, H1N1, rH1N1 or H1N2. The respective challenge control groups (E, F and G) excreted high titers of the challenge viruses for 5–6 days (mean AUC = 23.7, 25.1 and 23.6, respectively). In contrast, pH1N1-immune pigs showed complete protection (AUC = 0) against challenge with the homologous virus (group A) or with H1N1 (group B), and nearly complete protection against challenge with rH1N1 (group C) (mean AUC = 0.1). Virus excretion was detectable in 3 out of 5 pigs from group C, for 1 day only and at minimal virus titers. A slightly weaker protection was observed after challenge with H1N2 (group D): 4 out of 5 pigs had virus shedding for 1–3 days (mean AUC = 2.1).

Prior to the start of the experiment, pigs were seronegative against all tested influenza viruses in HI, VN and NI assays. At 2 weeks post-primary inoculation with pH1N1, pigs from groups A, B, C and D had similar antibody titers (*p* > 0.05) against the homologous virus in all assays. The geometric mean titers (GMTs) are shown in Table 3. At 6 weeks post-primary inoculation (time of the secondary inoculation with various H1 viruses), the HI, VN and NI GMTs against pH1N1 were 39, 106 and 260, respectively. Cross-reactive antibodies against H1N1, rH1N1 and H1N2 were undetectable in all pH1N1-immune pigs in the HI assay, but most pigs had low cross-reactive VN titers. Higher cross-reactive NI titers were detected against H1N1 (GMT 36) than against rH1N1 (GMT 17) (*p* < 0.05). The challenge control pigs (groups E, F and G) remained seronegative before the secondary inoculation, but had developed HI, VN and NI antibodies against the respective challenge virus at 14 days post-secondary inoculation (Table 3). Figure 2B illustrates the more rapid development of VN antibodies against the challenge virus in challenge control pigs than in pH1N1-immune pigs. Anti-pH1N1 antibody titers remained at pre-challenge levels in group A, B and C in all assays (*p* > 0.05), but increased significantly in group D in HI and VN assays (*p* < 0.05) (Additional file 3).

### Experiment 2: protection against challenge with a European H3N2 SIV in pigs with infection immunity against various H1 virus(es)

**Figure 3:**
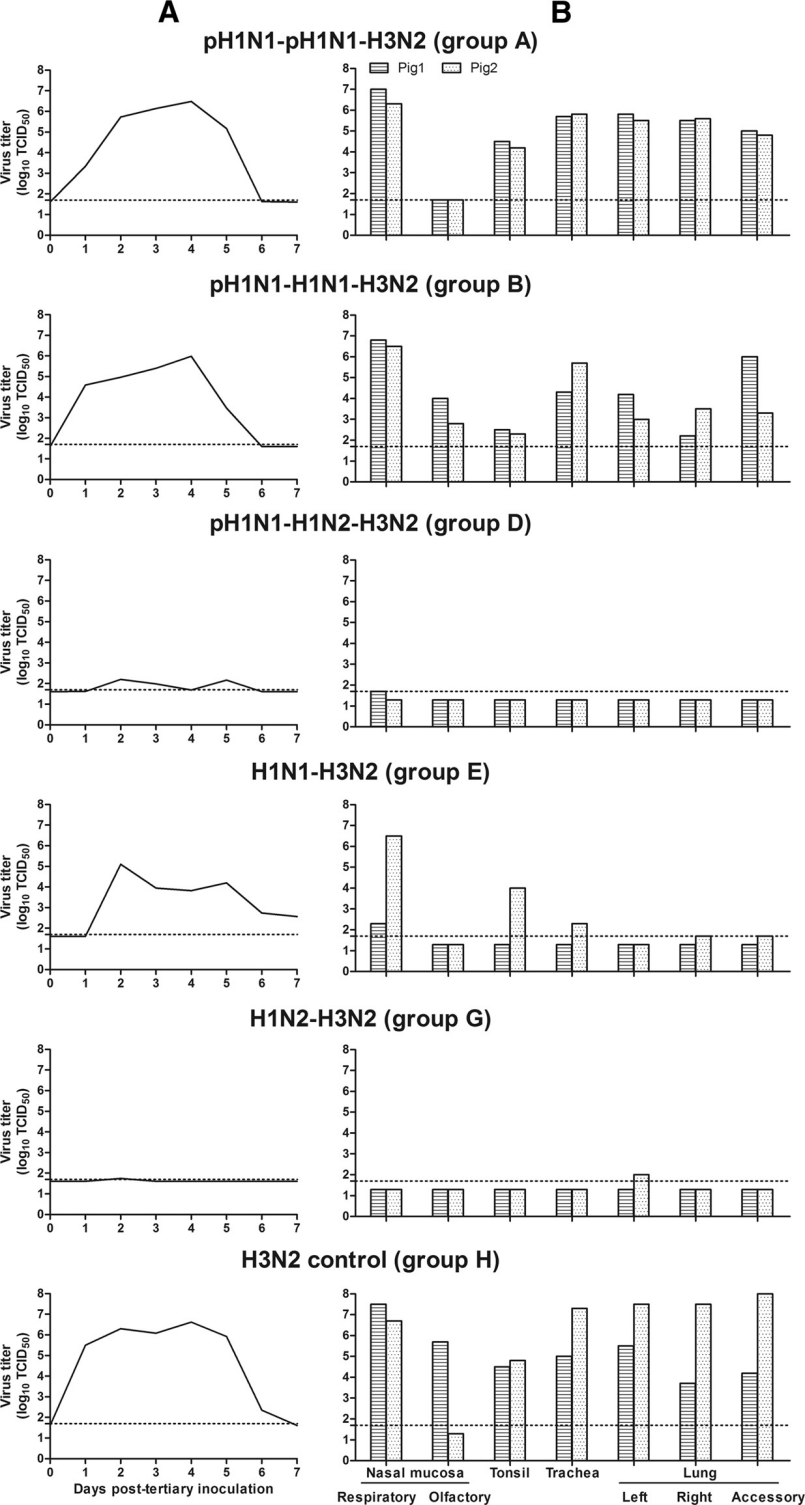
**Virus titers in nasal swabs (A) and respiratory tissues (B) post-tertiary inoculation with H3N2.** Nasal swabs were collected daily from day 0 to 7 post-tertiary inoculation to determine virus titers per 100 mg nasal secretions. Two pigs per group were euthanized at day 4 post-tertiary inoculation to determine virus titers in 1 g respiratory tissues. Horizontal dotted lines represent the detection limit for virus titration: 1.7 log_10_ TCID_50_.

Figure 3A shows mean virus titers in nasal swabs post-tertiary inoculation with a European H3N2 SIV. All challenge control pigs (group H), pigs immune to pH1N1 (group A) or pH1N1 followed by H1N1 (group B) shed high titers of virus for 4–6 days (mean AUC = 23.2, 18.9 or 16.4, respectively). All H1N1-immune pigs (group E) also shed viruses for at least 2–6 days, but the virus titers were reduced (mean AUC = 12.3). Only one pig with infection immunity against H1N2 (group G) and two pigs with infection immunity against both pH1N1 and H1N2 (group D) had detectable virus excretion (mean AUC = 0.14 and 1.65, respectively). Figure 3B shows individual virus titers in the respiratory tract of 2 pigs of each group at 4 days post-tertiary inoculation. Challenge control pigs (group H) were virus-positive in all tissues, except for the olfactory region of the nasal mucosa of one pig. Virus isolation rates and virus titers in the other groups reflected those in nasal swabs. Pigs immune to pH1N1 (group A) or both pH1N1 and H1N1 (group B) showed only a minimal reduction of virus replication. A higher reduction of virus titers was observed in the pigs immune to H1N1 alone (group E), while those immune to H1N2 alone (group G) or pH1N1 followed by H1N2 (group D) were almost completely protected against H3N2 replication.

All pigs lacked HI and VN antibodies against H3N2 before the tertiary inoculation. Cross-NI antibodies against H3N2 were only detected in pigs previously exposed to H1N2, and titers were higher in group G (GMT 70) than in group D (GMT 17) (*p* < 0.05). At 14 days post-tertiary inoculation with H3N2, all pigs developed antibodies or showed an increase in pre-existing antibody titers against H3N2 (Table 4). Antibody titers against H1 viruses remained at pre-challenge level in all assays (*p* > 0.05).

**Table 4:** **Geometric mean antibody titers against the H3N2 virus in hemagglutination-inhibition (HI), virus-neutralization (VN), and neuraminidase-inhibition (NI) assays at 14 days post-tertiary inoculation with H3N2**

Group	Virus inoculations	HI	VN	NI
A	pH1N1-6w-pH1N1-17w-H3N2	202	116	320
B	pH1N1-6w-H1N1-17w-H3N2	254	266	254
D	pH1N1-6w-H1N2-17w-H3N2	16	26	320
E	Mock-6w-H1N1-17w-H3N2	127	185	320
G	Mock-6w-H1N2-17w-H3N2	20	16	320
H	Mock-6w-mock-17w-H3N2	160	347	381

## Discussion

We have shown nearly complete cross-protection against replication of European H1 SIV lineages in pigs with infection immunity against pH1N1, but only a weak cross-protection against the H3N2 subtype in pigs with infection immunity against various H1N1 viruses. In line with previous studies [9,27], all three H1 SIVs used in our studies failed to cross-react with pH1N1 in HI assays with post-infection swine sera, and there was minimal cross-reactivity in VN assays. The VN assay is known to be more sensitive than the HI assay, and the former assay does not only detect antibodies that inhibit the attachment of the virus to target cells, but also antibodies that can block the fusion of the viral and endosomal membranes [28]. Cross-HI assays with high-titered hyperimmune swine sera have also shown low levels of cross-reactivity between pH1N1 and European avian-like H1N1 but not human-like H1N2 SIVs [27]. The HA1 of the European H1 SIVs had similar low percentages (69-71%) of amino acid homology to pH1N1. However, a detailed analysis of the antigenic sites revealed fewer amino acid differences between the classical H1 of pH1N1 and the avian-like H1 of H1N1 as compared to the human-like H1 of rH1N1 and H1N2 SIVs. Interestingly, the Sa (13 amino acids) site is an immunodominant antigenic site [29-31], and was found to be completely conserved between the first two viruses as previously reported [32], while there were 5–6 amino acid differences between pH1N1 and the human-like H1 SIVs. This may explain why the pH1N1-immune pigs in our study were best protected against the avian-like H1N1 SIV. A complete cross-protection against the pH1N1 after prior infection of pigs with the avian-like H1N1 SIV has been demonstrated in a previous study [9]. Likewise, prior infection or vaccination with a 1918 pandemic or classical swine H1N1 virus, which differ from pH1N1 in only 1 amino acid in the Sa antigenic site, resulted in nearly complete protection from the latter virus in mice and ferrets [33-35]. Furthermore, there is strong evidence for cross-protection between pH1N1 and historical human seasonal H1N1 viruses from the 1930-40s in humans and in experimental animal models [34,36,37]. Yet, these viruses have only 67-76% amino acid homologies in their HA1 and as many as 5 amino acid differences in the Sa antigenic site [34]. Some of the viruses used for prior infection and challenge in the present study also have NAs of the same lineage. This was the case for pH1N1 and European SIVs of the H1N1 subtype (H1N1 and rH1N1), as well as for the European H1N2 and H3N2 SIVs. Unlike antibodies against HA, anti-NA antibodies cannot neutralize influenza viruses, but they do play a significant secondary role in protection against influenza in humans and animals [38-41]. Therefore, it is not surprising that pH1N1-immune pigs were better protected against European H1N1 than H1N2 SIVs, and that H1N2-immune pigs were better protected against H3N2 challenge than H1N1-immune pigs.

Protection between viruses of H1N1 and H3N2 subtype was clearly less robust than that between viruses of the same HA and/or NA subtype. Yet, pigs with infection immunity against the avian-like H1N1 SIV were better protected against H3N2 than pH1N1-immune pigs. This suggests a contribution of immune responses against internal proteins, which are shared between avian-like H1N1 and human-like H3N2 SIVs but are of different origins in pH1N1, except for the M protein. In pigs that had been subsequently exposed to pH1N1 and the avian-like H1N1, protection against H3N2 was similar to that in pigs exposed twice to pH1N1, and inferior to that in pigs exposed to H1N1 alone. This is most likely due to the failure of the H1N1 virus to replicate and induce specific immune responses in pH1N1-immune pigs, as indicated by the nasal virus titers and serological responses. Our data are largely in agreement with previous studies on heterosubtypic protection in pigs, ferrets, and mice [11,13-16,42], none of which showed a complete protection between H1 and H3 viruses. For example, prior infection of ferrets with pH1N1 or a seasonal H1N1 virus resulted in a shorter duration of nasal excretion of seasonal H3N2 virus, i.e. 5 days instead of 7 days in unprimed influenza naïve ferrets [13,15]. In mice, prior infection with H1N1 or pH1N1 did not reduce H3N2 virus titers in the lungs at 4 days post-challenge, but titers were undetectable or reduced by 10^4^-fold at 7 days post-challenge [11,42].

Although heterosubtypic protection has been frequently studied in mice, the pig model has some specific advantages for studying protection between influenza viruses. Pigs are natural hosts for a variety of genetically and antigenically diverse H1 and H3 viruses. Most SIVs have an HA derived from viruses that once circulated in humans and the pH1N1 is a shared virus between humans and swine [43]. Nearly all SIVs are natural reassortants. As a result, the SIVs used in this study share one to seven genes with each other, and this allows examination of the relative importance of the corresponding proteins for protection. As pointed out before [44-46], the pathogenesis of influenza is very similar in pigs and in humans, and there are also striking similarities in their immune responses. In mice, the outcome of infection experiments depends on the mouse and virus strain used. For instance, upon infection with PR/8, DBA/2 mice showed a greater susceptibility to infection, more rapid weight loss and death, elevated cytokine production, and more severe lung histopathology than C57BL/6 mice [47]. Also, human influenza viruses generally require adaptation to be able to replicate and achieve virulence in mice, and the adapted virus may be antigenically and phenotypically very different from the initial strain [48]. Finally, mice transmit influenza viruses inefficiently and nasal virus excretion cannot be evaluated in mice [49]. Pigs, in contrast, are highly susceptible to a variety of human H1 and H3 viruses [24,50,51]. And unlike mice, pigs can shed high titers of virus in nasal swabs for 4–6 days.

During the last few years, there have been several serologic investigations for cross-reactive antibodies against European H1 and H3 SIVs in humans of different age categories [52-54]. HI antibodies against the European H3N2 SIV were present in 70% of those born before 1990, but were rare in the younger population [54]. In comparison, HI antibodies against the European avian-like H1N1 SIV were detected in only approximately 10% of humans in 2009, and they showed only a minimal increase in humans with seroconversion to pH1N1 [53,55,56]. This is in agreement with our finding that pH1N1 infection did not induce detectable serum HI antibodies against the avian-like H1N1 SIV. On the other hand, pH1N1-immune pigs showed a complete virological protection against the avian-like H1N1 SIV. This further supports the notion that infection with a live influenza virus can induce cross-protection in the absence of cross-reactive serum HI antibodies and argues for more in vivo cross-protection studies in animal models. From such studies, we conclude that humans, especially the young population born after 1990, will likely have better immune protection against European H1 than H3 SIVs. This is based on the presence of minimal protection against European H3N2 SIVs in pigs with infection immunity against contemporary human H3N2 viruses [24] or pH1N1, whereas infection immunity against pH1N1 seems to offer significant protection against the major H1 SIVs.

In conclusion, our study shows that infection with a live, wild type influenza virus may offer substantial cross-lineage protection against viruses of the same HA and/or NA subtype. Heterosubtypic protection between viruses of different HA and NA subtypes, in contrast, appears to be weak in pigs. Because they are natural hosts for the same influenza virus subtypes as humans, pigs have some unique advantages as a model for cross-protection studies with influenza. According to our data, the global spread of pH1N1 in humans will enhance their cross-protective immunity against European H1 SIVs, making those viruses less likely to cause pandemics in the near future.

## Additional files

Additional file 1:
**Alignment of deduced amino acid sequences in the HA1 of A/California/04/09 (pH1N1), sw/Gent/28/10 (H1N1), sw/Côtes d’Armor/0046/08 (reassortant H1N1), and sw/Gent/26/12 (H1N2).** Residues in the open boxes represent previously identified antigenic sites of H1. Amino acids differing from those in the A/California/04/09 sequence are shown, conserved residues are represented as dots. Additional file 2:
**Alignment of deduced amino acid sequences in the NA of sw/Gent/172/08 (H3N2) and sw/Gent/26/12 (H1N2).** Residues in the open boxes represent previously identified antigenic sites of N2. Amino acids differing from those in the sw/Gent/172/08 sequence are shown, conserved residues are represented as dots. Additional file 3:
**Serological profile after inoculation with various H1 viruses in pH1N1-immune pigs.** Antibody titers were determined at 14 days post-secondary inoculation in hemagglutination-inhibition (HI), virus-neutralization (VN), and neuraminidase-inhibition (NI) assays.
