# Supplementary figures and images for: Cross-protection against European swine influenza viruses in the context of infection immunity against the 2009 pandemic H1N1 virus: studies in the pig model of influenza
Source: Vet Res. 2015 Sep 24;46:105. doi: 10.1186/s13567-015-0236-6 (PMC4581489; doi:10.1186/s13567-015-0236-6)

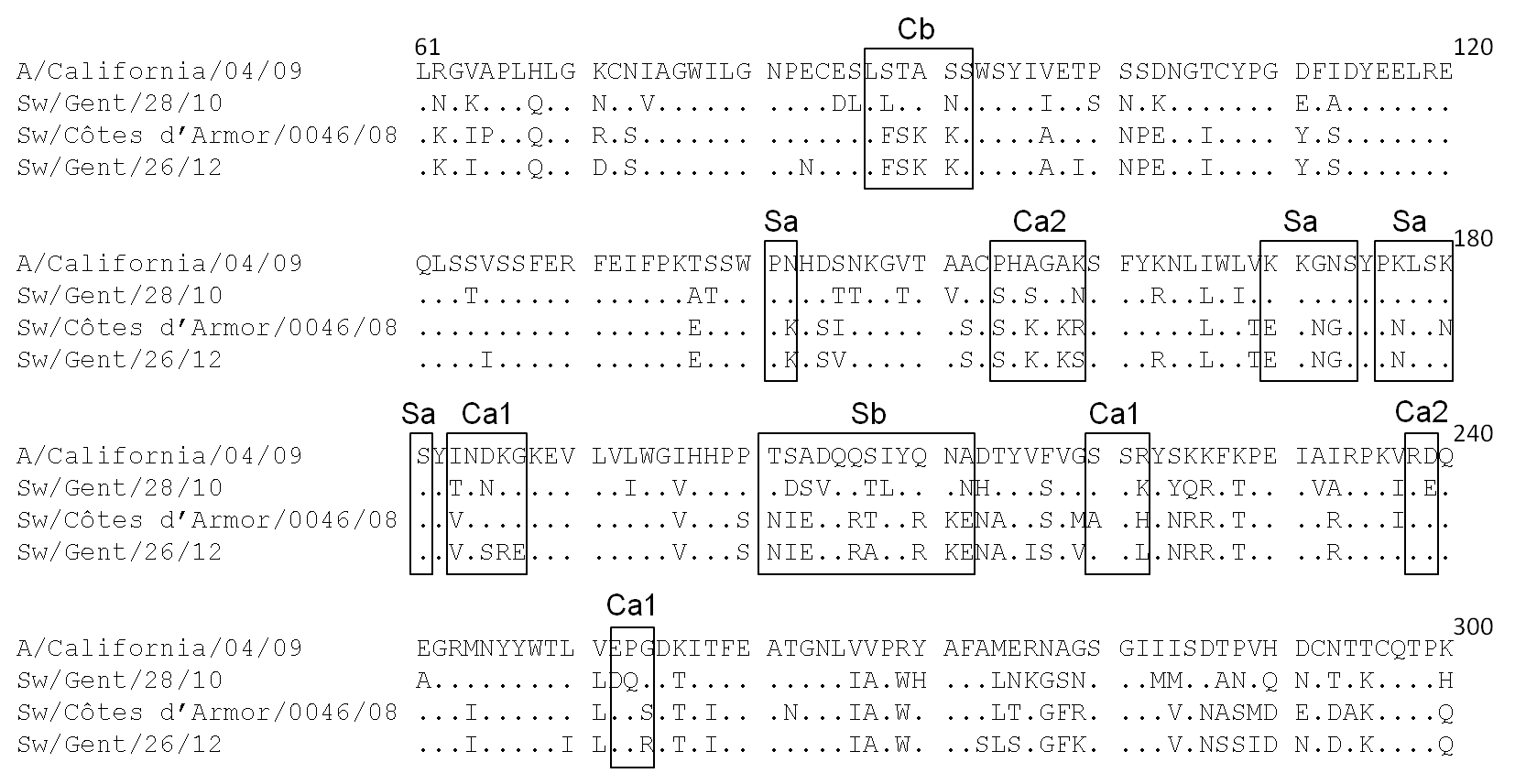

Supplement: Additional file 1: — Alignment of deduced amino acid sequences in the HA1 of A/California/04/09 (pH1N1), sw/Gent/28/10 (H1N1), sw/Côtes d’Armor/0046/08 (reassortant H1N1), and sw/Gent/26/12 (H1N2). Residues in the open boxes represent previously identified antigenic sites of H1. Amino acids differing from those in the A/California/04/09 sequence are shown, conserved residues are represented as dots. [file 13567_2015_236_MOESM1_ESM.tif]

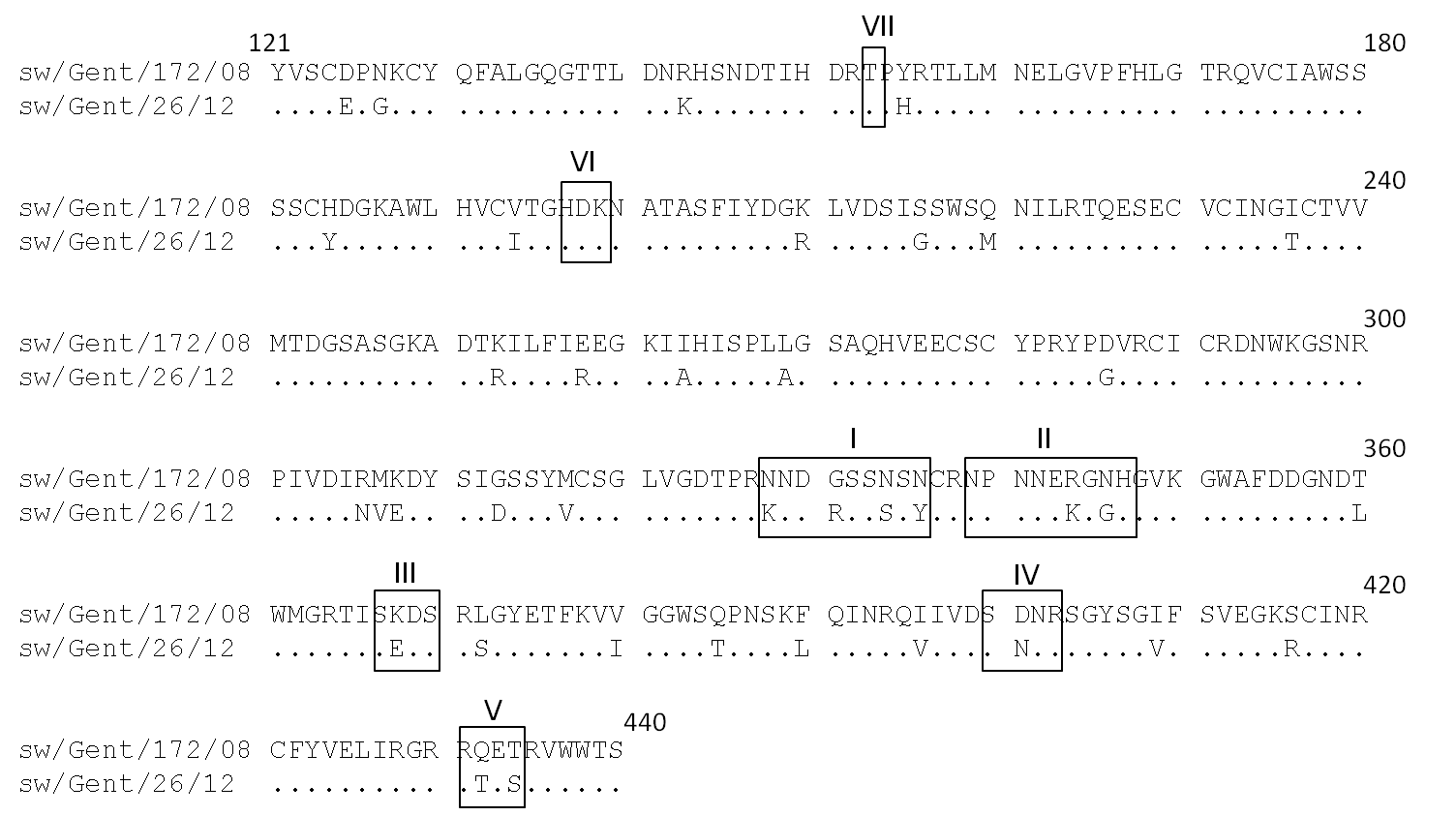

Supplement: Additional file 2: — Alignment of deduced amino acid sequences in the NA of sw/Gent/172/08 (H3N2) and sw/Gent/26/12 (H1N2). Residues in the open boxes represent previously identified antigenic sites of N2. Amino acids differing from those in the sw/Gent/172/08 sequence are shown, conserved residues are represented as dots. [file 13567_2015_236_MOESM2_ESM.tif]
