# Supplementary material for: Cross-protection against European swine influenza viruses in the context of infection immunity against the 2009 pandemic H1N1 virus: studies in the pig model of influenza
Source: Vet Res. 2015 Sep 24;46:105. doi: 10.1186/s13567-015-0236-6 (PMC4581489; doi:10.1186/s13567-015-0236-6)
Supplement: Additional file 3: — Serological profile after inoculation with various H1 viruses in pH1N1-immune pigs. Antibody titers were determined at 14 days post-secondary inoculation in hemagglutination-inhibition (HI), virus-neutralization (VN), and neuraminidase-inhibition (NI) assays. [file 13567_2015_236_MOESM3_ESM.docx]

|  |  | No. of pigs positive for antibodies (geometric mean antibody titer of the positive pigs) | | | | | | | | | | | | | | |
| --- | --- | --- | --- | --- | --- | --- | --- | --- | --- | --- | --- | --- | --- | --- | --- | --- |
|  |  | A/California/04/09 (pH1N1) | | |  | Sw/Gent/28/10 (H1N1) | | |  | Sw/Côtes d’Armor/0046/08 (rH1N1) | | |  | Sw/Gent/26/12 (H1N2) | | |
| Group | Virus inoculations | HI | VN | NI |  | HI | VN | NI |  | HI | VN | NI |  | HI | VN | NI |
| A | pH1N1- 6w-pH1N1 | 5 (53) | 5 (131) | 5 (184) |  | 0 | 4 (5) | 5 (17) |  | 0 | 5 (7) | 5 (13) |  | 0 | 5 (16) | 0 |
| B | pH1N1-6w-H1N1 | 5 (80) | 5 (228) | 5 (485) |  | 2 (10) | 5 (41) | 5 (139) |  | 0 | 5 (9) | 5 (53) |  | 0 | 5 (12) | 0 |
| C | pH1N1- 6w-rH1N1 | 5 (26) | 5 (142) | 5 (368) |  | 0 | 5 (10) | 5 (53) |  | 1 (10) | 5 (58) | 5 (40) |  | 0 | 5 (53) | 0 |
| D | pH1N1-6w-H1N2 | 5 (106) | 5 (1536) | 5 (485) |  | 0 | 5 (47) | 5 (139) |  | 5 (17) | 5 (602) | 5 (92) |  | 5 (23) | 5 (346) | 5 (23) |
